# Determinants of Behavioral Intention and Use of Interactive Whiteboard by K-12 Teachers in Remote and Rural Areas

**DOI:** 10.3389/fpsyg.2022.934423

**Published:** 2022-06-17

**Authors:** Ying Zhou, Xinxin Li, Tommy Tanu Wijaya

**Affiliations:** ^1^School of Mathematics and Statistics, Guangxi Normal University, Guilin, China; ^2^School of Mathematical Sciences, Beijing Normal University, Beijing, China

**Keywords:** IWB, UTAUT2, learning media, educational technologies, behavior intension

## Abstract

Interactive Whiteboard (IWB) has recently been used to replace the TWB (traditional whiteboard), with many of its features being observed to help teachers in educational activities. This is based on effectively and efficiently increasing the teacher-student interaction. Therefore, this study aims to analyze the determinants of Behavioral Intention (BI) and the use of interactive whiteboards by K-12 teachers, in remote and rural Chinese areas. The Modified-Unified Theory of Acceptance and Use of Technology 2 (UTAUT2) model was used in this analysis, as a learning medium to deliver the subject matter to students. The sample and population were also the teachers in the Guangxi Zhuang Autonomous Region, China, where 171 voluntary respondents participated in this study. Furthermore, the obtained data were processed using a Structural Equation Model (SEM) approach, through the Smart-PLS software. The results showed that Habit and Hedonic Motivation had a significant influence on the Behavioral Intention (BI) of teachers, toward the utilization of IWB in remote and rural areas. Besides this, Facilitating Conditions (FCs) and BI also had a significant positive effect on Usage Behavior. Based on these results, important information was provided to school principals, local governments, and teachers for education quality improvement, regarding the patterns of increasing IWB utilization in remote and rural areas.

## Introduction

All factors are found to be influenced by information technology in the 21st Century, including the education sector. In developing countries, the government also focuses on improving the quality of education in rural and remote areas, which is one of the main priorities of the Ministry of Education in China (Aixia et al., 2020; Huang et al., 2020). In this condition, many methods have been developed by this educational sector, such as providing training to each school to improve teacher pedagogical and technological knowledge (Qin et al., 2019; Chai et al., 2020). This indicates that information technology is included in teaching and learning activities, and creates a rich educational environment with various technological media. Improving the quality of education in rural and urban areas proves that the main goal of the government is to ensure the technological integration of all teaching and learning activities in the regional educational field (Liu et al., 2021). Based on the Chinese government data, the use of digital learning media was found to be less than 50%, which is a utilization challenge for regional principals (Huang et al., 2020). Ministry of Education of the People’s Republic of China (2018) also stated that the use of technology in learning activities was closely related to the improvement of students’ abilities, subsequently revealing that school principals need to critically consider the use of these media in every classroom. They also need to remind teachers and provide training on the utilization of technology in teaching and learning activities. One of these learning media is based on the use of Interactive Whiteboard (IWB), which is still not optimally utilized by the teacher when transferring knowledge to students (Morgan, 2010; Shi et al., 2021). To effectively utilize the Interactive Whiteboard, the analysis of the most important influential usage factors is highly necessary.

IWB is one of the potential technology-based media used to improve the quality of learning activities when the embedded features are maximally utilized (Šumak and Šorgo, 2016). However, the main problem emphasizes the suitable influential factors used for the Behavioral Intention (BI) and usage attitude of teachers in remote and rural areas (Pujiastuti and Haryadi, 2019). Increasing the use of IWB is also difficult in these regions, especially the maximization of educational activities. Based on many previous studies, several differences were observed in the perceptions and attitudes of urban and rural teachers, toward the use of technology-based learning media (Kim and Jung, 2010; Siaw et al., 2018; Niens et al., 2021). Some of them also showed the factors influencing these teachers’ utilization intentions, with the integration of IWB in poor rural and remote areas found to be limited (Setyono and Cahyo, 2017; Siaw et al., 2018; Shao and Lee, 2020). This verifies that a subsequent report is still needed to obtain a deeper understanding of the models and methods responsible for the factors influencing teachers toward using IWB in remote and rural areas. Therefore, this study aims to determine and predict the potential factors influencing the Behavioral Intention and attitude of the teachers using IWB in remote and rural areas. This is carried out by using the Unified Theory of Acceptance and Use of Technology (UTAUT2) model (Venkatesh et al., 2012), with the results being expected to help the regional government and schools toward the broad utilization of IWB. The formulation of the study problems is shown as follows,

• What factors influence the Behavioral Intention of K-12 teachers to use IWB in remote and rural areas?
• What is the biggest significant positive factor influencing the Behavioral Intention of teachers in remote and rural areas, based on using IWB in teaching and learning activities?
• What factors influence the K-12 teachers’ usage behaviors in using IWB within remote and rural areas?
• What is the biggest significant positive factor influencing the Usage Behavior of K-12 teachers in remote and rural areas, based on the utilization of IWB in teaching and learning activities?

## Literature Review

### Interactive Whiteboard

**FIGURE 1:**
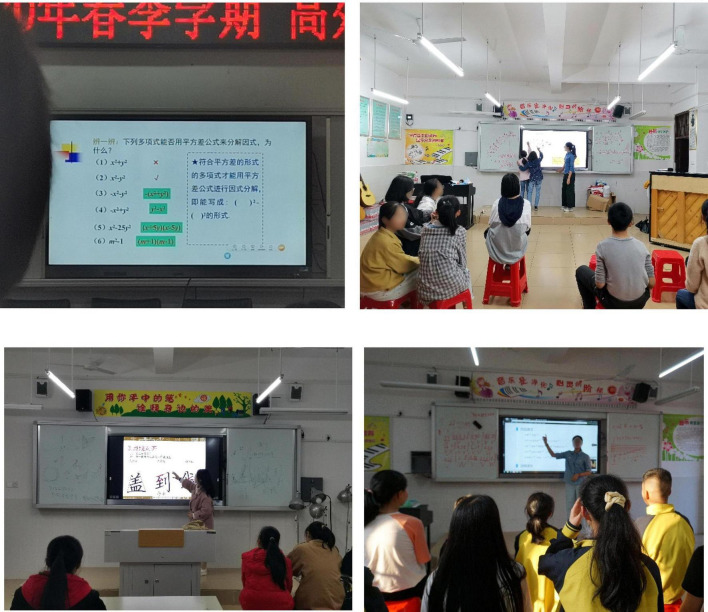
The IWB supporting interactive teaching methods in the classroom.

Interactive whiteboard (IWB) has reportedly shown significant changes and feature additions in recent years, with the first version being a virtual electronic of the traditional blackboard (Türel, 2011; Bardakci and Alkan, 2019). This is widely used in important events, as in conferences, and in virtual classrooms, to observe the written or graphical knowledge being gained by the teachers or students. Meanwhile, the second latest version combines computer, microelectronics, and electronic communication technologies, to display PowerPoint, interactive learning media, micro-games, or micro-lectures (Morgan, 2010; Shi et al., 2021). It is also equipped with a pen to enable teachers to write important points on the IWB screen when providing new materials to students. In this study, the utilized samples are the schools having the second version of IWB, which has many features to support interactive teaching methods. Each class is also equipped with a tablet, computer, IWB, and Internet connection, for the adequate integration of technology in teaching and learning activities, as shown in Figure 1.

Many previous studies showed that IWB was recognized by various teachers as one of the technology-based presentation and learning media, which had many advanced and easily-operated features to improve the quality of educational activities (Campbell and Kent, 2010; Šumak and Šorgo, 2016). Using classroom IWB, a Singapore study on 124 preservice teachers showed increased learning involvement and activeness (Divaharan and Koh, 2010). This was in line with a qualitative study, where the utilization of the learning media increased student engagement (Timothy and Sharon, 2013; Shi et al., 2021). Furthermore, IWB facilitates and enhances the teaching effect of teachers, with many studies revealing an effective increase and decrease in learning impacts and burdens, respectively, due to the combination of appropriate educational methods and strategies (Alsawaie and Alghazo, 2010; Bardakci and Alkan, 2019). The University of Virginia Center for Teacher Education and Technology also found that the learning media based on technology helped in maintaining the coherence, reduction, and elevation of thinking, stress, and confidence, respectively (Juersivich et al., 2009). learning media based on technology also improved students’ creativity, understanding, and mastery of teaching content (Sautière et al., 2019; Desania et al., 2020; Chen et al., 2021; Suryawan et al., 2021). In addition, a good visual presentation effect on IWB effectively improved students’ cognition and understanding of teaching content in the learning process (Morgan, 2010). These conditions confirmed that the learning media were suitable for utilization in remote and rural areas, where teachers have low technological knowledge. Despite this, Shi et al. (2021) argued that not all traditional whiteboards were still likely to be replaced with IWB in future. Many studies also showed the effectiveness of IWB when used to deliver teaching materials, although only a few examined the factors influencing teachers’ usage behaviors (Türel, 2011; Shi et al., 2021). This indicated that the effectiveness of IWB was unable to be maximized without understanding the influential factors.

### The Situation of Interactive Whiteboard in Remote and Rural Areas of China

The Chinese government shows great importance to the development of educational information technology in remote and rural areas, due to the numerous visitations to observe the quality of education (Hu et al., 2016). This is because the adopted policy advocacy is based on the provision of materials, technology, teachers, and other resources, to promote the development of education in these areas (Ministry of Education of the People’s Republic of China, 2021). In recent years, the government strengthened classroom construction and equipped teaching sites with appropriate technology-based equipment, in response to the weak development of schools (Ministry of Education of the People’s Republic of China, 2017; Zhu, 2019). These pieces of equipment included campus networks, IWB, physical booths, audio, and cameras. This was subsequently carried out by promoting equal educational distribution and improving regional teaching quality through technology integration. In this case, many international studies showed that the application of information technology had certain positive effects on education and teaching (Wijaya et al., 2020, 2022; Weinhandl et al., 2021). According to a regional study in Chinese remote and rural areas, the integration of technology into educational activities provided a new atmosphere and experience for students, which affected increasing learning interest and Motivation (Wang et al., 2018; Zhou and Li, 2020). However, several problems, such as the reluctance of teachers to use digital learning media were based on the non-maximization of institutional technology integration (Peng et al., 2014). Some Chinese remote and rural area reports also showed that information technology had specific educational teaching values, although several problems are often observed such as the utilization reluctance of teachers.

Interactive whiteboard is one of the easiest digital learning media utilized by novice teachers and in urban-rural areas (Campbell and Martin, 2010). This is observed as an early beginning before the integration of other classroom technologies, to improve active learning compared to conventional teaching methods (Campbell and Martin, 2010; Mun et al., 2019). It also helps teachers to display interesting animations and use interactive digital textbooks, micro-games, micro-lectures, and other teaching methods capable of increasing students’ interest and Motivation (Türel and Demirli, 2010; Šumak and Šorgo, 2016; Shi et al., 2021). In addition, many previous studies showed that IWB was widely used as one of the most basic technology-based learning media, which improved teacher innovation and creativity (Türel, 2011).

### The Unified Theory of Acceptance and Use of Technology (UTAUT-2) and Study Hypothesis

**FIGURE 2:**
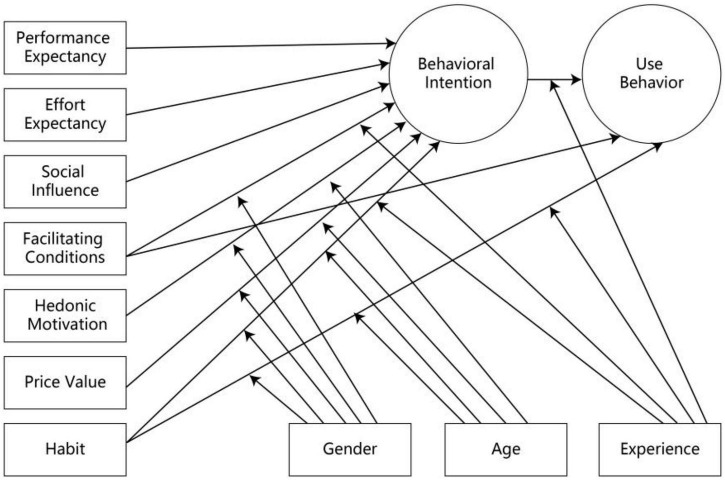
UTAUT-2 MODEL (Venkatesh et al., 2012).

Based on previous reviews, technological acceptance theories and models were developed to explain and analyze the factors influencing users’ intentions toward new technologies. According to Davis (Venkatesh et al., 2003), the Technology Acceptance Model (TAM) was proposed, to primarily explain and predict the use of information technology through the factors of performance and effort expectancies. Subsequently, many models were created through continuous revision and expansion, to observe the factors using new technologies, such as the TRA (Theory of Reasoned Action) (Fishbein and Ajzen, 1975), MPCU (Innovation Diffusion Theory and Model of Personal Computer Utilization) (Fang and Liu, 2017), TPB (Theory of Planned Behavior) (Cheng, 2017), IDT (Innovation Diffusion Theory) (Rogers, 1995), SCT (Social Cognitive Theory) (Bandura, 1986), C-TAM-TPB (TAM Model with extension Model TPB), and MM (Motivational Model) (Bagozzi et al., 1992). Based on these theoretical models, Venkatesh et al. (2003) also integrated the UTAUT- (Unified Theory of Acceptance and Use of Technology), which analyzed 2 dependent variables, namely BI (Behavior Intention) and UB (Usage Behavior). These were then influenced by 4 core variables, namely PE (Performance Expectancy), EE (Effort Expectancy), SI (Social Influence), and FCs (Facilitating Conditions). To subsequently expand the applicability and explanatory power of the UTAUT model, Venkatesh et al. (2012) then proposed the UTAUT-2 model (Figure 2). From the variables in the UTAUT model, this new method also provided three core variables, namely, Hedonic Motivation (HM), Price Value (PV), and Habit (HB). Although the UTAUT-2 model was proposed in 2012, it is still widely used to explain user factors toward new technologies. Moreover, some studies directly used the UTAUT-2 model as a basic method or a combination with other approaches, where core or extensive variables are observed (El-Masri and Tarhini, 2017; Choi and Hoo-Jo Hoog, 2018; Abdul Rabu et al., 2019; Cao and Nguyen, 2022). Besides the consideration of the innovative technology acceptance, the UTAUT-2 model also emphasizes the usage behavior of the user (Faqih and Jaradat, 2021). Based on previous studies, the fundamental difference between both models focused on the applicability of UTAUT by creating new structures and new relationships (Moorthy et al., 2019).

UTAUT is considered to be more suitable and useful for analyzing the factors within the use of new educational technology (Mashroofa et al., 2019; Lavidas et al., 2022). This explained that the UTAUT and UTAUT-2 models were used to analyze user intentions in the education sector, information systems, e-commerce, and other technological fields (Tan, 2013; Cheung et al., 2018; Garg, 2021). Under these conditions, several experimental topics included (1) e-learning platforms (Alismaiel, 2021), (2) distance education platforms (Nikou, 2021), (3) multimedia learning-space stations, and (4) online communities (Liao et al., 2016). These studies indicated that when the use of urban-rural IWB became a habit, its effectiveness in improving students’ abilities was subsequently enhanced. However, McLean (2005) showed that teachers were very reluctant to use a new teaching technology in these areas. This led to the need to analyze the factors influencing teachers to integrate new technologies in the rural and remote areas, especially IWB. Under these conditions, UTAUT-2 was used to predict technological adoption to approximately 70% (Gellerstedt et al., 2018), compared to the TAM model whose explanation was observed at 40% (Drueke et al., 2021). Another reason for using the UTAUT-2 model was that no other suitable method has analyzed the utilization of new educational technologies. To compare the TAM and UTAUT models, empirical tests were also carried out, with the UTAUT being more suitable for utilization in educational fields (Abd Rahman et al., 2021).

Due to government investment, the remote and rural area schools installed free-to-use interactive electronic whiteboards, indicating that Price Value did not influence teachers in utilizing IWB. This led to the removal of PV. Besides, although the UTAUT model was more stable and widely used to predict the users’ intention (UI) of new technology utilization, the early related techniques were developed from digital and business worlds. This caused the reset of some constructs and moderators when used to predict the usage intentions in other fields (Dwivedi et al., 2019). These observations were not in line with analyses of some experts, which argued that UTAUT-2 moderating factors, such as age, gender, and experience had no effect on the UI of new educational technology usage (Dwivedi et al., 2019; Chatterjee and Bhattacharjee, 2020; Abbad, 2021). However, it was supported by several studies, where the model’s moderator effect had no significant effect on the educational sector (Liebenberg et al., 2018; Bardakci and Alkan, 2019).

The UTAUT-2 model is divided into the following variables:

1. *Independent Variables:* PE (Performance Expectancy), EE (Effort Expectancy), SI (Social Influence), FC (Facilitating Condition), HM (Hedonic Motivation), and HB (Habit).
2. *Intermediate Variable:* Behavioral Intention (BI)
3. *Dependent variable:* Usage Behavior (UB)

The six independent variables were found to casually affect the use of interactive electronic whiteboards in remote and rural areas, as comprehensively observed as follows,

(1) Performance Expectancy (PE)

Performance Expectancy is defined as the degree to which individuals believe that new technology helps to improve their job performances. According to Venkatesh (Venkatesh et al., 2003), a significant positive effect of PE was observed on a person’s intention to adopt new technology (Wu et al., 2021). This indicated that the notion of this variable emphasized the extent to which frontline primary and secondary school teachers believed that IWB helped them improve their teaching performances. In addition, some reports confirmed that PE had a direct impact on the behavioral intentions of teachers to use technology (Han and Conti, 2020; Faqih and Jaradat, 2021; Shiferaw et al., 2021), due to the most important positive influential factor (Tan, 2013).

(2) Effort Expectancy (EE)

Effort Expectancy is the level of ease associated with the use of information technology (Li and Zhao, 2021). In this report, EE is the level of teachers’ efforts to use IWB in imparting new knowledge to the students in remote and rural areas. Based on previous studies, a significant positive effect of EE was observed on the BI to use new technology (Chatti and Hadoussa, 2021; Kim and Lee, 2020; Lulin et al., 2020). However, it was unclear whether the characteristics of the remote-rural teachers were in line with those in cities.

(3) Social Influence (SI)

Social Influence is the influence of people around the user, which indicates that the new technology should be used (Venkatesh et al., 2003). In previous reports, a positive effect of SI was observed on behavioral intentions (Isaias et al., 2017; Mujalli et al., 2022), although it was critically unclear whether rural and remote teachers trusted the surrounding people’s advice. Therefore, in this study, the impact of SI is defined as the acceptance level of teachers’ technology used by key figures (leaders or colleagues).

(4) Facilitating Conditions (FCs)

Facilitating Conditions are the teacher’s complete perception of the various technical support conditions needed for the successful use of information technology (Venkatesh et al., 2003). According to several previous reviews, significant positive effects of FCs were observed on UB and BI (Chao, 2019; Alowayr, 2021; Raffaghelli et al., 2022). Based on these results, this variable is the perception of frontline K-12 teachers in using IWB, regarding the convenience of use, equipment and technical support, and after-sale services, such as networking facilities and skills training. However, the Regional Chinese Government provided complete facilities to support the integration of technology in learning activities. This predicted that a significant positive effect of FC should be observed on teachers’ BI and UB to use IWB.

(5) Hedonic Motivation (HM)

Hedonic Motivation is the sense of fun and pleasure felt by the users when integrating new technology (El-Masri and Tarhini, 2017). When an individual feels a high level of happiness and satisfaction for being involved in an activity, the Behavioral Intention to use the new technology is found to become stronger (Cao and Nguyen, 2022). This was in line with many studies, where a significant positive effect was observed on the UB of technology-based learning media (Choi and Hoo-Jo Hoog, 2018; Faqih and Jaradat, 2021; Taamneh et al., 2022). The use of IWB media in remote and rural areas is also innovative as it is a new experience for teachers and students, which potentially leads to a sense of fun to utilize the information technology. In this study, HM is then defined as the belief of remote-rural teachers that the use of IWB enabled a sense of happiness or enjoyment, during teaching and learning activities.

(6) Habit (HB)

Habit is an additional variable in UTAUT-2, which has the strongest significant positive factor influencing the UB to use technology (Dahri et al., 2021; Prasetyo et al., 2021). This was in line with the study by Arain et al. (2019) and Moorthy et al. (2019), where a significant positive influence was observed on Usage Behavior. Under these conditions, the government provided IWB facilities to rural and remote schools, indicating that technology utilization has become a habit for a small group of teachers during educational activities. Therefore, this report defined HB as the teaching behavior of remote and rural teachers, who often use interactive electronic whiteboards.

(7) Behavior Intention (BI)

Behavior Intention influences an individual’s decision whether to use new technology in the future, with several previous studies showing a relationship between BI and UB (Prasetyo et al., 2021; Yu et al., 2021). This indicated that the variable was a direct determinant of actual behavior (Gao and Deng, 2012). In this analysis, BI is mainly defined as the willingness of frontline primary and secondary school teachers to use IWB during educational activities.

(8) Usage Behavior (UB)

Usage Behavior is the actual use of IWB by frontline primary and secondary school teachers during teaching processes.

**FIGURE 3:**
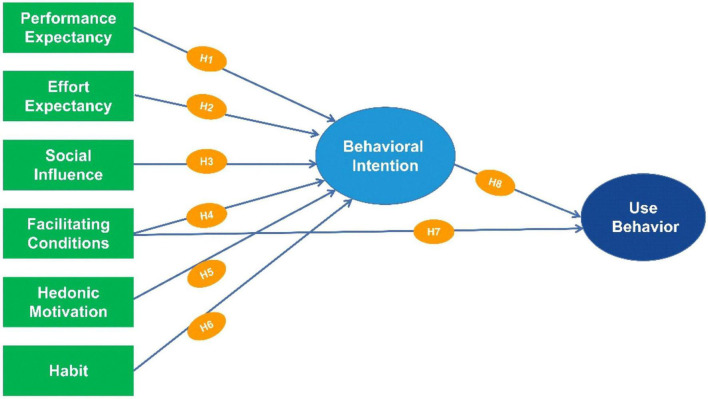
Proposed model based on UTAUT-2 (Venkatesh et al., 2012).

Based on these descriptions, the influential relationships between 6 independent, 1 intermediate, and 1 dependent variable were combined into 8 hypotheses, as shown in Table 1 and Figure 3.

**TABLE 1:** Study hypotheses.

Hypothesis number	Hypothesis content
H1	Performance expectancy has a positive effect on the BI of teachers to use IWB in rural and remote areas
H2	Effort expectancy has a positive effect on the BI of teachers to use IWB in rural and remote areas
H3	Social influence has a positive effect on the BI of teachers to use IWB in rural and remote areas
H4	Facilitating conditions have a positive effect on the BI of teachers to use IWB in rural and remote areas
H5	Hedonic Motivation has a positive effect on the BI of teachers to use IWB in rural and remote areas
H6	Habit has a positive effect on the BI of teachers to use IWB in rural and remote areas
H7	Facilitating Conditions have a positive effect on the UB of teachers to use IWB in rural and remote areas
H8	Behavior Intention has a positive effect on the UB of teachers to use IWB in rural and remote areas

## Study Methodology

Based on the influential factors, a quantitative approach was used to test the hypotheses of this report (Shukla, 2021). The UTAUT-2 model was also used to determine the BI and UB of teachers in China’s remote and rural areas, Guangxi Zhuang Autonomous Region. These areas are located in the western part of China, which is an underdeveloped area with relatively poor economic, technological, and educational levels (OECD, 2016; Xu et al., 2021). A purposive sampling technique was also used to obtain data from February to March 2022, through an online questionnaire filled out by teachers at the remote and rural schools in Guangxi, China. Before the collection of data, permission and informed consent were obtained from all teachers and schools willing to participate in this experiment. In addition, data were obtained through the online Wenjuanxing application, and disseminated through principals and teachers using email, WeChat, and QQ.

### Respondent

The objects were the primary and secondary school teachers in China’s remote and rural areas, such as Guangxi Zhuang Autonomous Region. Under these conditions, a valid sample of 171 people participated in the study, with questionnaire distribution adopting a combination of the online and on-site methods due to the pandemic. This was conducted to ensure the efficiency and quality of the data instrument recovery. According to Gorusch (1983), the sample size was related to the problem of measurement and respondents’ proportion, with the ratio being approximately 1:5. Furthermore, a total of 8 core variables with 24 measurement items were observed, with the sample size not expected to be less than 120 respondents, according to the principle of proportion. This indicated that a total of 180 questionnaires were distributed and returned, as 171 of them were valid with an effective rate of 95%, which met the requirements of data analysis. The basic information of these valid samples is shown in Table 2.

**TABLE 2:** Basic information of respondents.

Items	Description	*N*	Percentage
Gender	Male	68	39.77%
	Female	103	60.23%
Age	20–25 years	48	28.07%
	26–30 years	21	12.28%
	31–35 years	7	4.09%
	36–40 years	10	5.85%
	41–46 years	28	16.37%
	46 years	57	33.33%
Teaching experience	1–5 years	68	39.77%
	6–10 years	10	5.85%
	11–15 years	4	2.34%
	16–20 years	7	4.09%
	> 21 years	82	47.95%
Level education	Elementary school	50	29.24%
	Junior high school	89	52.05%
	Senior high school	32	18.71%
Learning subject	Mandarin language	38	22. 22%
	English	14	8.19%
	Mathematics	77	45.03%
	Biology	7	4.09%
	Chemical	5	2.92%
	Physics	1	0.58%
	Geography	2	1.17%
	History	6	3.51%
	Citizenship	8	4.68%
	Music	1	0.58%
	Art	3	1.75%
	Sport	2	1.17%
	Information and Communication Technology	3	1.75%
	Other	4	2.34%
Monthly use of technology-based interactive learning media	Never	25	14.62%
	1–2 times	43	25.15%
	3–4 times	24	14.04%
	5–6 times	26	15.2%
	> 7 times	53	30.99%
The level of IWB use as an interactive learning media	Beginner	76	44. 44%
	Intermediate	86	50.29%
	Expert	9	5.26%

### Instrument Development

The questionnaire contained two parts as follows: (1) the demographic information of respondents, including gender, age, teaching experience and period, subject, weekly utilization frequency of IWB, interactive smart whiteboards proficiency, and other basic information and (2) a UTAUT-2 questionnaire to determine the BI and Usage Behavior of IWB for remote and rural teachers. A total of 24 measurement items were also observed for PE, EE, SI, FC, HM, HB, BI, and UB, as they were adopted from previous reports and modified according to these experiments. Appendix Table 1 comprehensively shows all the measurement models and sources, with the second part of the questionnaire adopting a five-point Likert scale, to assign a score of 1, 2, 3, 4, and 5 for SD, D, N, A, and SA which represent strongly disagree, disagree, neutral, agree, and strongly agree, respectively.

### Measurement Scale

The Smart-PLS software and Structural Equation Modeling (SEM) were used to test the proposed model and evaluate the statistical significance of the path coefficients. According to Hair et al. (2016), the advantages of PLS-SEM were based on hypothetical analyses when the distribution and sample size are not normal and small, respectively. The evaluated measurement model also reflected and analyzed the reliability and validity of the construct, with the structural evaluation being used to identify the hypotheses (Alvi, 2021; Leow et al., 2021). Firstly, the reliability and validity of the MM (measurement model) were tested, with the ICR (Internal consistency reliability), and CV (convergent validity) being used for the item analysis. Secondly, the structural model was analyzed by statistical evaluation methods, such as the VIF value, path coefficient, t-statistics, and *P*-value, with the influential factors of each variable being explored (Alturki and Aldraiweesh, 2022). In addition, the *t*-test was used to compare the actual behavior of teachers in China’s remote and rural areas, regarding the use of IWB to carry out learning activities.

## Data Analysis and Results

To analyze the overall situation of the questionnaire and the effect of all independent variables, descriptive statistics were initially used to test the normality of the data. This indicated that the mean score, standard deviation, kurtosis, and other basic information were derived from the 24 measurement items (Lei and Lomax, 2005). The measurement model, reliability, validity, and collinearities model analyses were also carried out in this study. Subsequently, the R2 model, effect size, and 8 hypotheses were analyzed.

### Descriptive Statistics and Normality Test of Measurement Items

Table 3 shows that the highest and lowest average scores of the items were HM3 and SI3 with 4.047 and 3.468, respectively. This proved that most teachers believed in some games, music, animation, and other functions of the IWB, which enabled more class fun. From the mean dimensions (PE = 3.98, EE = 3.79, SI = 3.74, FC = 3.71, HM = 3.99, HB = 3.73, BI = 3.91, and UB = 3.63), most of the teachers provided positive answers, with an average score of 3.91 and 3.63 in BI and UB, respectively. This revealed that they were optimistic about the willingness and behavior of using the blackboard. According to previous studies (Hair et al., 2012), the normality test of the data was | 1|, with the skewness and kurtosis appropriately within the range of acceptable values. This verified that all data were normally distributed.

**TABLE 3:** Descriptive statistics.

Items		Mean statistic	Standard deviation statistic	Excess kurtosis statistic	Skewness statistic
Performance Expectancy	PE1	4.018	0.914	−0.015	−0.637
	PE2	4.023	0.917	0.707	−0.872
	PE3	3.895	0.924	−0.012	−0.595
Effort Expectancy	EE1	3.725	0.872	0.109	−0.446
	EE2	3.766	0.900	−0.454	−0.295
	EE3	3.877	0.880	0.166	−0.536
Social Influence	SI1	3.865	0.837	0.368	−0.645
	SI2	3.895	0.866	−0.170	−0.449
	SI3	3.468	1.136	−0.661	−0.294
Facilitating Conditions	FC1	3.778	0.996	−0.042	−0.616
	FC2	3.737	1.000	0.019	−0.582
	FC3	3.626	0.967	−0.357	−0.321
Hedonic Motivation	HM1	3.965	0.830	0.753	−0.677
	HM2	3.959	0.868	0.352	−0.625
	HM3	4.047	0.884	0.297	−0.707
Habit	HB1	3.801	0.959	−0.435	−0.394
	HB2	3.713	0.901	−0.189	−0.318
	HB3	3.690	0.957	−0.264	−0.352
Behavior Intention	BI1	3.901	0.863	−0.338	−0.357
	BI2	3.930	0.869	−0.225	−0.456
	BI3	3.895	0.859	−0.207	−0.410
Usage Behavior	UB1	3.661	0.974	−0.366	−0.345
	UB2	3.602	1.017	−0.624	−0.281
	UB3	3.637	1.013	−0.404	−0.380

### Measurement Model Evaluation

The analysis of the instrument’s reliability and validity was carried out using the Smart-PLS 3.0 software. This showed that the convergent validity was verified from the measurement model, by testing the item and composite reliabilities, average variance extracted (AVE), and Cronbach’s alpha (Hair et al., 2016).

Table 4 confirms that the Cronbach’s alpha, composite reliability, and AVE values of the 8 latent variables ranged from 0.818 to 0.949, 0.891–0.962, and 0.735–0.908, which exceeded the standard 0.7, 0.7, and 0.5, respectively (Hair et al., 2016). These satisfactory results indicated that the study scale was very reliable. Meanwhile, the loading factor of all items was greater than 0.5 in the validity test, which subsequently met the standard. For discriminant validity, the criteria Fornell and Larcker (1981) were adopted, using the square root of AVE for each latent variable. Table 5 shows that the diagonal element of the AVE square root (numbers in bold) was greater than the other correlation values among other latent variables, verifying that discriminant validity was established between the constructs of this scale.

**TABLE 4:** Loading factor, validity, and reliability.

Latent variable	Indicator	Loading	T-value	Cronbach’s alpha	Composite reliability	Average variance extracted (AVE)
Performance Expectancy	PE1	0.944	64.387	0.928	0.954	0.873
	PE2	0.927	50.669			
	PE3	0.932	66.486			
Effort Expectancy	EE1	0.872	27.352	0.883	0.928	0.811
	EE2	0.895	45.092			
	EE3	0.933	84.820			
Social Influence	SI1	0.916	67.953	0.818	0.891	0.735
	SI2	0.922	71.925			
	SI3	0.717	11.963			
Facilitating Conditions	FC1	0.883	32.023	0.885	0.929	0.812
	FC2	0.919	60.719			
	FC3	0.902	53.122			
Hedonic Motivation	HM1	0.953	111.435	0.923	0.951	0.867
	HM2	0.940	85.760			
	HM3	0.900	36.966			
Habit	HB1	0.904	39.942	0.905	0.940	0.840
	HB2	0.920	66.324			
	HB3	0.925	64.241			
Behavioral Intention	BI1	0.951	89.921	0.949	0.967	0.908
	BI2	0.958	90.027			
	BI3	0.949	89.606			
Usage Behavior	UB1	0.937	76.088	0.934	0.958	0.883
	UB2	0.940	67.521			
	UB3	0.941	77.217			

**TABLE 5:** Fornell-Larcker criterion.

	PE	EE	SI	FC	HM	HB	BI	UB
PE	**0.934**							
EE	0.806	**0.901**						
SI	0.803	0.760	**0.857**					
FC	0.697	0.695	0.693	**0.901**				
HM	0.775	0.687	0.747	0.718	**0.931**			
HB	0.748	0.754	0.734	0.761	0.772	**0.916**		
BI	0.750	0.672	0.734	0.709	0.827	0.799	**0.953**	
UB	0.646	0.706	0.645	0.657	0.660	0.852	0.742	**0.939**

*PE, performance expectancy; EE, effort expectancy; SI, social influence; FC, facilitating conditions; HM, hedonic motivation; HB, habit; BI, behavioral intention; UB, use behavior.*

### Collinearity Test

A certain correlation was also observed among the model variables, which led to the necessity for a collinearity test in regression analysis (Latan and Noonan, 2017). This was conducted to exclude the reciprocal effect observed among the independent variables (Al-rahmi et al., 2021). Furthermore, VIF (Variance Inflation Factor) is the ratio of the variance when multicollinearity is present and absent between the explanatory variables, with the linear relationship being analyzed among all the independent factors (B. Wu, 2021). This proved that the VIF value was preferable, accepted, and strongly collinear with < 5 and 10, and > 10, respectively.

Table 6 shows that the latent variable of the model did not have strong collinearity (VIF < 5), indicating the following: (1) the items of the 7 constructs did not overlap, (2) each item independently reflected the indicators to be measured, and (3) the setting of the questionnaire was reasonable.

**TABLE 6:** The VIF values of the internal model.

	Behavioral intention	Usage behavior
Performance expectancy	4.384	
Effort expectancy	3.588	
Social influence	3.542	
Facilitating conditions	2.828	2.010
Hedonic motivation	3.454	
Habit	3.718	
Behavioral intention		2.010

### Structural Model Evaluation

After the measurement model, a structural model assessment was conducted by calculating the coefficient of determination (R^2^) and SRMR, path coefficient, and effect size (Latan and Noonan, 2017; Mulyani et al., 2021). The significance level of 5% and run bootstrapping were also used for size variations through the Smart-PLS software (Latan and Noonan, 2017). Moreover, the hypothetical analysis was determined by the value of the t-statistic and *p*-value, with results being obtained after bootstrapping calculations (Figure 3). This confirmed that the inner and outer model values were the path coefficient/internal T-values and the weight/external T-values, respectively.

**FIGURE 4:**
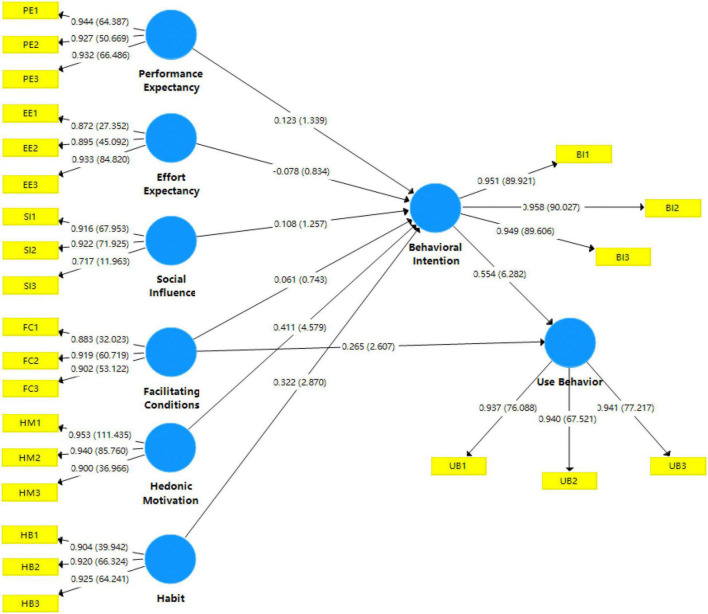
The structural model with inner and outer models.

Based on Figure 4, the loading factors of the eight variables (outer model) were observed between 0.872 and 0.958, which are greater than the standard 0.7 (Kline, 2005). This indicated that the observations adequately explained the underlying variables. To evaluate the explanatory power of the model variables, the coefficient of determination (R^2^) was also used (Vidergor, 2021). According to previous studies, R^2^ values greater than 0.67, and between 0.33–0.67 and 0.19–0.33 were considered to be high, moderate, and weak, respectively (Chao, 2019; Abbad, 2021). In this report, the R^2^ values of BI and UB were 0.761 and 0.585, indicating a high and moderate explanatory power (76.1% and 58.5%), respectively.

The Standardized Root-Mean Square Residuals (SRMRs) are appropriate for assessing the suitability of the PLS model (Koh et al., 2013), where a good fit is often defined when the SRMR value is less than 0.10 (Luik et al., 2018). This confirmed that the SRMR value of 0.047 < 0.1 which depicted that the proposed model had a strong fit.

### Hypothesis Testing

**FIGURE 5:**
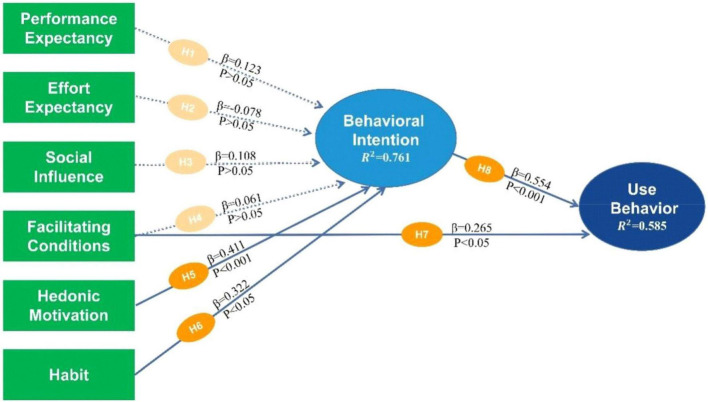
Path coefficient and *p*-values.

The assumptions of the model’s latent variables were subsequently tested, especially the path coefficient, t-value, and *p*-value, to verify the hypotheses of the theoretical method and empirical data, as shown in Table 7 and Figure 5.

**TABLE 7:** Hypothesis analysis.

Hypothesis	Relationship	Path coefficient (B)	Sample mean	Standard deviation (STDEV)	t Statistic	*p* values	Decision
H1	Performance Expectancy - > Behavioral Intention	0.123	0.125	0.092	1.339	0.181	Refuse
H2	Effort Expectancy - > Behavioral Intention	−0.078	−0.077	0.093	0.834	0.404	Refuse
H3	Social Influence - > Behavioral Intention	0.108	0.112	0.086	1.257	0.209	Refuse
H4	Facilitating Conditions - > Behavioral Intention	0.061	0.059	0.083	0.743	0.458	Refuse
H5	Hedonic Motivation - > Behavioral Intention	0.411	0.412	0.090	4.579	0.000	Supported
H6	Habit - > Behavioral Intention	0.322	0.317	0.112	2.870	0.004	Supported
H7	Facilitating Conditions - > Usage Behavior	0.265	0.268	0.102	2.607	0.009	Supported
H8	Behavioral Intention - > Usage Behavior	0.554	0.552	0.088	6.282	0.000	Supported

Table 7 shows the path coefficient, sample-mean standard deviation, t-statistics, and the level of significance (*p*-value). This proved that H5, H6, H7, and H8 had t-statistics > 1.96 and *p*-values < 0.05, subsequently indicating that they had a significant effect.

Based on these results, PE, EE, SI, and FC had no positive effects on BI by using IWB (β = 0.12, *t* = 1.34 < 1.96, *p* = 0.18 > 0.05; β = −0.09, *t* = 0.834 < 1.96, *p* = 0.40 > 0.05; β = 0.108, *t* = 1.26 < 1.96, *p* = 0.21 > 0.05; and β = 0.06, *t* = 0.74 < 1.96, *p* = 0.46 > 0.05), indicating the rejection of H1, H2, H3, and H4, respectively. Meanwhile, HM and HB had positive effects on BI by using IWB (β = 0.41, *t* = 4.58 > 1.96, *p* = 0.00 < 0.05 and β = 0.32, *t* = 2.87 > 1.96, *p* = 0.00 < 0.05), leading to the acceptance of H5 and H6, respectively. For Usage Behavior (UB), FC and BI were also observed to have positive effects by using IWB (β = 0.265, *t* = 2.607 > 1.96, and *p* = 0.01 < 0.05 and β = 0.55, *t* = 6.28 > 1.96, and *p* = 0.00 < 0.05), verifying the acceptance of H7 and H8, respectively.

Table 8 summarizes the direct, indirect, and total effects of each variable on the behavioral intention and usage behavior of remote and rural teachers, toward the use of IWB during learning activities.

**TABLE 8:** The direct, indirect, and total effects in the path model.

Factor	Determinant	Effect		
		Direct	Indirect	Total
Behavior Intention = 0.761	PE	0.123	0	0.123
	EE	−0.078	0	−0.078
	SI	0.108	0	0.108
	FC	0.061	0	0.061
	HM	0.411	0	0.411
	HB	0.322	0	0.322
Usage Behavior = 0.585	PE	0	0.068	0.068
	EE	0	−0.043	−0.043
	SI	0	0.060	0.060
	FC	0.265	0.034	0.299
	HM	0	0.228	0.228
	HB	0	0.178	0.178
	BI	0.554	0	0.554

Table 8 shows that the determinants with the greatest and second-largest influences on BI were HM and HB at total effect values of 0.411 and 0.322, respectively. Meanwhile, the two main factors affecting UB were BI and FC, at direct effect values of 0.554 and 0.299, respectively.

## Discussion and Implications

Using the UTAUT-2 model, this study aimed to identify the factors influencing the behavioral intention and usage behavior of K-12 teachers on the utilization of IWB. It also determined the strongest significantly-positive effect on teachers’ BI and UB by using IWB learning media.

### The Effect of Hedonic Motivation on Behavioral Intention

From the results, HM highly focused on the enjoyment of remote and rural K-12 teachers regarding the usage of IWB as a very interesting application. Based on their beliefs, certain games, music, animations, and other IWB functions increased the classroom fun and ensured students’ happiness during educational activities. Furthermore, HM was observed as the most powerful independent variable in the BI of these teachers, although no previous scholarly reports were found (Martins et al., 2018; Moorthy et al., 2019). These results were supported by a previous ICT study, where the HM of teachers’ information-technology use had a positive impact (Choi and Hoo-Jo Hoog, 2018; Nikolopoulou et al., 2021). This led to the following implications: (1) software developers need to increase the interest and pleasure of the application during the design of the functions, for the teachers to utilize them more and (2) managers such as schools and education bureaus should train teachers toward using IWB to develop interesting teaching games.

### The Effect of Habit on Behavioral Intention

Habit was observed and predicted to have a significant positive and moderate effect on K-12 teachers’ behavioral intention to use IWB. This showed that the variable led the teachers’ BI toward using the learning tool in teaching processes, exhibiting similarities to previous consistent studies (Nikolopoulou et al., 2021; Taamneh et al., 2022). Therefore, good habits were important for helping teachers adapt to the use of information technology in the new era, where, the utilization of educational network platforms has become a common learning method (Taamneh et al., 2022). Some previous studies explained that when students or teachers have poor habits in using these platforms (Martins et al., 2018; Moorthy et al., 2019; Taamneh et al., 2022), they eventually quit due to a lack of self-control in the learning process. Although teachers are active builders, they presently have insufficient initiatives as they are still more accustomed to using traditional blackboards for educational activities. Therefore, schools need to provide teachers with conditions for using IWB, while helping them develop active habits by setting indicators, such as a check-in system, point reward, and teaching assessment.

### The Effect of Facilitating Conditions on Usage Behavior

Facilitating conditions predicted had significant positive and moderate effects on teachers’ Usage Behavior, although no influence was observed on their Behavioral Intentions toward using information technology in the classroom. Despite these results, Facilitating Conditions variable still affected the utilization of IWB, which showed consistency with the study of Ma et al. (2019). This indicated that most of the remote and rural K-12 teachers were not proficient in using the learning media in educational activities. It also proved that 50% of them were over 35 years of age as indicated in Table 2, and were not very proficient in the use of technology to teach. This is because the educational resources in remote rural areas are not very good (Shao and Lee, 2020; Sulisworo et al., 2020), accompanied by unsystematic pieces of training and ignorance of institutional information technology. Although the educational information infrastructures in remote and poverty-stricken areas have been significantly improved, the occurrence of their utilization effects was still observed compared to relatively developed sites. In this condition, some schools did not even carry out special IWB training, although arranged for teachers to personally learn through online teaching videos. This seriously affected the information technology UB of primary and secondary school teachers in remote and rural areas. Therefore, decision-makers such as schools and the regional education department need to provide systematic and professional IWB-based skills to K-12 teachers in remote and poverty-stricken areas, to support teaching and learning activities.

### The Effect of Behavioral Intention on Usage Behavior

Behavioral Intention was the biggest predictor affecting the use of IWB by primary and secondary school teachers in remote and impoverished areas. This indicated that BI had a high-level impact on Usage Behavior, with thinking and action maintaining a high degree of consistency. These results were in line with many previous reports, where performances were always predicted when a strong desire and will were observed (Efiloğlu Kurt and Tingöy, 2017; Bardakci, 2019). Therefore, these underdeveloped teachers should be strengthened in future to cultivate the BI to use IWB, as well as HM and HB.

### Performance and Effort Expectancies and Social Influence Predictors Do Not Have a Significant Effect on Behavioral Intention

Based on the results, the BI and UB of K-12 teachers in remote and rural areas were not influenced by PE, EE, and SI. This was slightly different from other previous reports, as PE and EE were often significantly affected (Estriegana et al., 2019; Almaiah et al., 2021; Alturki and Aldraiweesh, 2022). Meanwhile, Social Influence was predicted to have an impact on technology in some studies (Maduku, 2017; Asghar et al., 2021). These differences were due to the particularity of the sample, as the objects in this study were underdeveloped K-12 teachers in remote and impoverished areas, with more comprehensive explanation as follows:

### Performance Expectancy Has No Significant Effect on Behavioral Intention and Usage Behavior

It was a surprise finding that PE was not significant to the BI and UB of K-12 teachers in remote and rural areas, regarding the use of IWB. This was not in line with many studies, which predicted that performance expectation was the strongest factor influencing users to use new technologies (Sánchez-Prieto et al., 2017; Garg, 2021). In this condition, the following explanations were determined: (1) teachers were located in remote and rural areas lacking educational resources and inadequate training. Besides this, they also believed that traditional classes with teacher-centered learning were more effective than using various technology-based media, (2) these teachers had low-performance expectations and assumed that IWB did not help improve the quality of their learning, and (3) some applications and learning media, such as micro-lectures and micro-games, were not suitable for operation on the IWB system. To overcome this problem, the schools and the regional government should jointly provide training and guidance to teachers in remote and rural areas, to increase learning effectiveness. Regarding students, the software developers also need to improve the system for efficient and effective performance.

### Effort Expectancy Has No Significant Effect on Behavioral Intention and Usage Behavior

Some K-12 teachers in remote and rural areas assumed that the operation of using IWB was more complicated in teaching and learning activities. Our finding was in line with the study by Ayaz and Yanartaş (2020), which predicted that EE did not have a significant impact on the use of educational technology. In this condition, the following explanations were determined: (1) the ease of use factor was insufficient to increase the usage intention of new educational technology and (2) the users were more willing to use this technology in the classroom based on the availability of adequate infrastructure, as well as related implementational training and tools.

### Social Influence Does Not Have a Significant Effect on Behavioral Intention and Usage Behavior

In this condition, teachers in remote and rural areas often assumed that their teaching methods were good and considered useless when using technology-based learning media. This was because approximately 50% and more of the respondents in this study were over 35 years of age with 10 years of teaching experience (Table 2). Regardless of whether they were recommended by colleagues, school leaders, or the neighborhood in remote and rural areas, IWB was still selected according to their preferences and habits. Furthermore, more than 50% of the respondents had teaching experience > 10 years, indicating that these teachers had their respective suitable habits and styles for classroom application. Based on these results, social influence did not have a significant effect on the intentions used in new learning media. This was in line with that reported by Nikolopoulou et al. (2021), who argued that SI was not related to the preference of teachers toward Information Technology utilization.

## Conclusion

To support government and school programs toward improving information technology integration in learning activities, especially in remote rural areas, the UTAUT-2 model was used to explore the factors influencing the Behavioral Intentions and Usage Behaviors of K-12 teachers. The data were analyzed using the smartPLS software, with hypothetical analysis being carried out through the partial least squares structural equation model (PLS-SEM). Of the 8 analyzed hypotheses, only HM/HB and FC/BI had significant positive effects on Behavioral Intentions and Usage Behaviors, respectively. Therefore, the government and schools should have practical and constructive solutions to increase the use of IWB in remote and rural areas. In this case, the principals and government need to provide teachers with training and design learning models, which have the capability of being combined with the use of IWB. Through the performance of some training processes, schools should also popularize the ease of using IWB and other technology-based interactive learning media. Therefore, these processes take a long time for teachers to believe that IWB has the ability to improve their learning performances. The government should also form a group or community capable of supporting teachers to use IWB in remote and rural areas. This group should be useful when the teachers have complaints or difficulties in using the learning media during the performance of educational activities. The community should also create social influence, toward the improvement of using IWB to teach. Considering continuous facilities and support from the government and schools, the use of technology-based learning media should essentially increase in remote and rural areas, especially the utilization of IWB.

## Limitations and Future Study

First, despite the results, several limitations were still observed, including the relatively small scope of the questionnaire obtained from the K-12 teachers, where the data obtained did not sufficiently represent all of them in China. Second, the data were only analyzed using a quantitative approach and a structural equation model. This indicates that subsequent reports need to use qualitative and quantitative methods to deeply explain the factors influencing the teachers’ BI and UB toward the utilization of IWB in teaching and learning activities. Third, future studies should extend UTAUT-2 and provide several moderator variables to affect the Behavioral Intention and Usage Behavior of teachers. Finally, comparative reports need to be carried out in rural and developed areas, to deepen the understanding of the analysis and different suggestions provided for each region.

## Data Availability Statement

The data analyzed in this study is subject to the following licenses/restrictions: the data will available by request. Requests to access these datasets should be directed to TW, 202139130001@mail.bnu.edu.cn.

## Ethics Statement

The studies involving human participants were reviewed and approved by Guangxi Normal University. The ethics committee waived the requirement of written informed consent for participation.

## Author Contributions

TW and XL conceived the study. YZ contributed to the supervision. XL conducted the experiment and collected the data. TW and YZ analyzed and interpreted the data and contributed to the writing of the manuscript. All authors have read and agreed to the published version of the manuscript.

## Conflict of Interest

The authors declare that the research was conducted in the absence of any commercial or financial relationships that could be construed as a potential conflict of interest.

## Publisher’s Note

All claims expressed in this article are solely those of the authors and do not necessarily represent those of their affiliated organizations, or those of the publisher, the editors and the reviewers. Any product that may be evaluated in this article, or claim that may be made by its manufacturer, is not guaranteed or endorsed by the publisher.

## Appendix

**TABLE A1:** Detailed questionnaire items.

Variable construct	Code	English version	Chinese version	Source
Performance Expectancy (PE)	PE1	IWB helped me to quickly complete my teaching preparation		Venkatesh et al., 2003
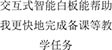
	PE2	IWB improved my teaching quality		
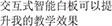
	PE3	Using IWB to teach improved my position in the school		
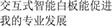
Effort Expectancy (EE)	EE1	It is easy for me to operate IWB to teach		Venkatesh et al., 2003
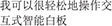
	EE2	Learning to use IWB is very easy		
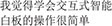
	EE3	Teaching using IWB is easier than traditional methods		
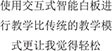
Social Influence (SI)	SI1	Other teachers recommended that I use IWB in teaching and learning activities		Venkatesh et al., 2003
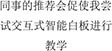
	SI2	The principal recommended that I use IWB when teaching		
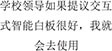
	SI3	Students recommended that I use IWB when teaching		
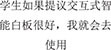
Facilitating Conditions (FC)	FC1	Local governments and schools conduct a lot of training on the use of IWB		Venkatesh et al., 2003
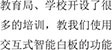
	FC2	Every class in the school has available IWB		
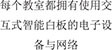
	FC3	There is a team that is ready to help when I have difficulties in using IWB		
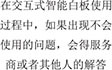
Hedonic Motivation (HM)	HM1	I think IWB is an interesting app		Venkatesh et al., 2012
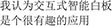
	HM2	Using IWB to teach is fun		
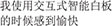
	HM3	Using IWB to teach is enjoyable		
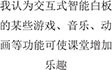
Habit (HB)	HB1	I am used to teaching through IWB		Venkatesh et al., 2012
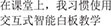
	HB2	I am addicted to teaching through IWB		
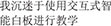
	HB3	I have to use IWB to teach		
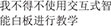
Behavioral Intention (BI)	BI1	I use IWB to teach in the future		Venkatesh et al., 2003
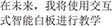
	BI2	I intensely use IWB to teach in the future		
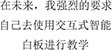
	BI3	I try to use IWB when teaching		
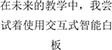
Use Behavior (UB)	UB1	I regularly use IWB to teach in class		Venkatesh et al., 2003
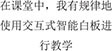
	UB2	Using IWB to teach is a pleasant experience		
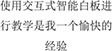
	UB3	I spend a lot of time on IWB use in my class.		
